# Removal of broken reamer stuck into femoral shaft in implanting PFNA: a case report

**DOI:** 10.1051/sicotj/2018024

**Published:** 2018-07-13

**Authors:** Gang-Qiang Jiang, Ya-Di Zhang, Yun-Qiang Zhuang

**Affiliations:** Department of Orthopedics, Ningbo NO. 6 Hospital, NO. 1059 ZhongShan Road, 315040 Ningbo PR China

**Keywords:** Broken reamer, Removal, Fermoral

## Abstract

Femoral intertrochanteric fracture is very common in elderly population. The usual treatment for these patients is intra-extramedullary fixation. In normal situations, expand medullary cavity is needed, in order to implant various intramedullary implants. On rare occasion, which will in turn lead to the reamer is stuck into the medullary cavity of femoral shaft. Open or closed technique for moving of the broken nails had been reported before. We firstly report a novel technique by using handy tool which included in orthopaedic instrument set to remove the broken reamer stuck into femoral cavity when implanting a PFNA.

## Introduction

Femoral   intertrochanteric fracture is very common in elderly population, especially with osteoporosis. The usual treatment for these patients is intra-extramedullary fixation. However, for most A2 to A3 (AO Classification) fractures, the intramedullary fixation may be the first option. In normal conditions, we should expand medullary cavity in order to implant various intramedullary implants. On rare occasion, which will in turn lead to the reamer is stuck into the medullary cavity of femoral shaft. And the result is, orthopaedic surgeons have to face the challenge of how to move the reamer without greater damaging femoral medullary cavity. Open or closed technique for moving of the broken nails had been reported before, nevertheless, moving a stuck steamer had few experience described. We firstly report a novel technique by using handy tool which included in orthopaedic instrument set to remove the broken reamer stuck into femoral cavity.

### Case presentation and surgical technique

**Figure 1:**
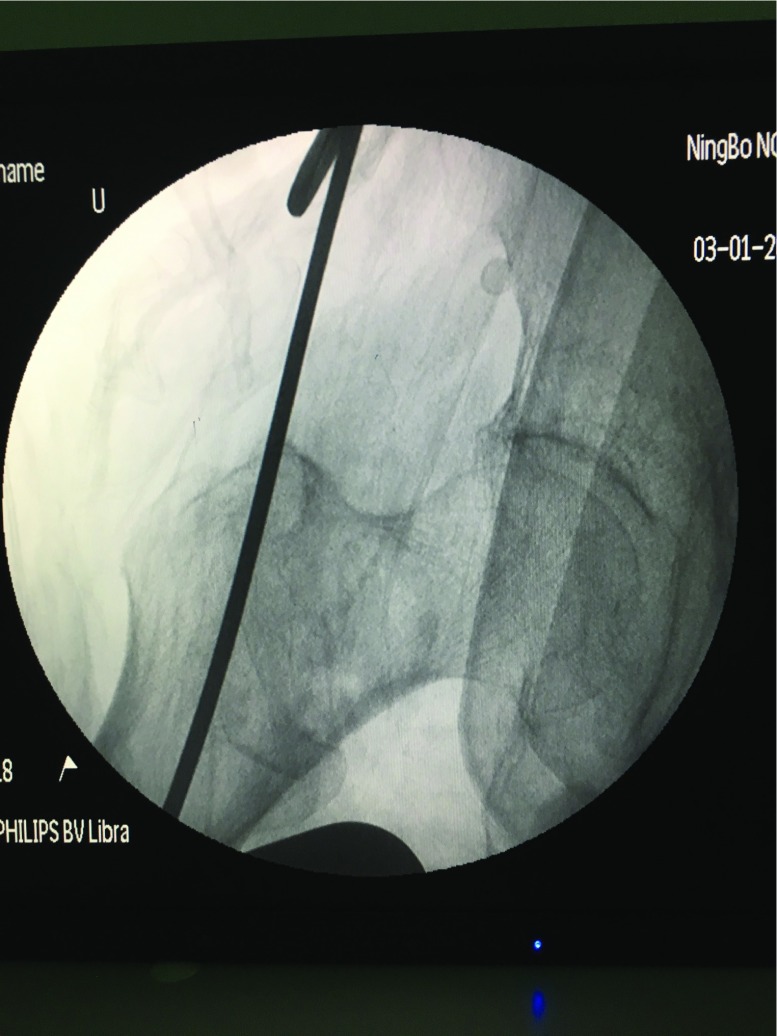
After reduction of intertrochanteric fracture, a guide pin was inserted into tip of the trochanter.

**Figure 2:**
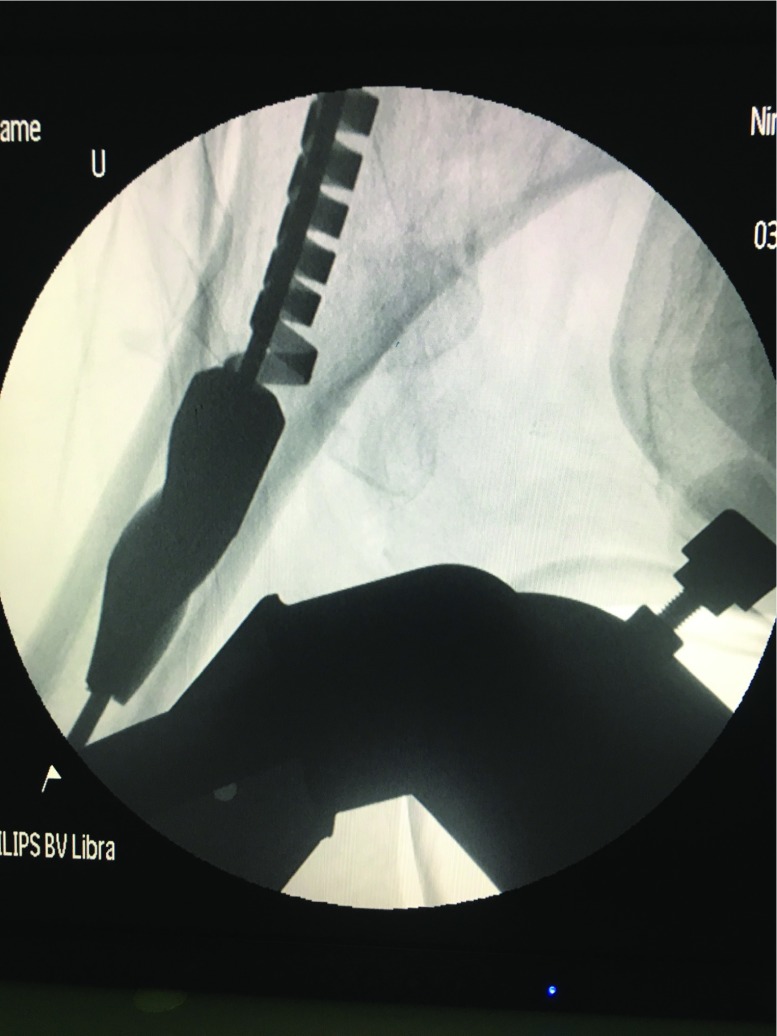
The reamer head was broken, then ruptured completely finally, and remained in femoral shaft isolated, it can’t pull out just rely on hauling.

An 80-year-old lady suffered a comminuted intratrochanteric fracture following a mechanical fall, with an AO classification of 3.1A2.3. After we excluded all contraindications, drew up a series of preoperation plans, consist of measure medullary cavity diameter, anterior femoral arch angle and the optimal entry point and so on. We treated with satisfied close reduction with traction on a traction operating table, and then captured the best point to insert the guide needle (Figure 1). In sequence, reamed medullary cavity was performed step-by-step. Unfortunately, the reamer was stuck into femoral medullary cavity tightly at last, we could not move out by traction or rotation. What's the worst, the reamer head was ruptured completely finally, and remained in femoral shaft isolated (Figure 2). The broken reamer located much more distal to the femoral intertrochanteric fracture site and jammed with cortex of bone firmly. So an extreme tough challenge for removal was in front of us.

**Figure 3:**
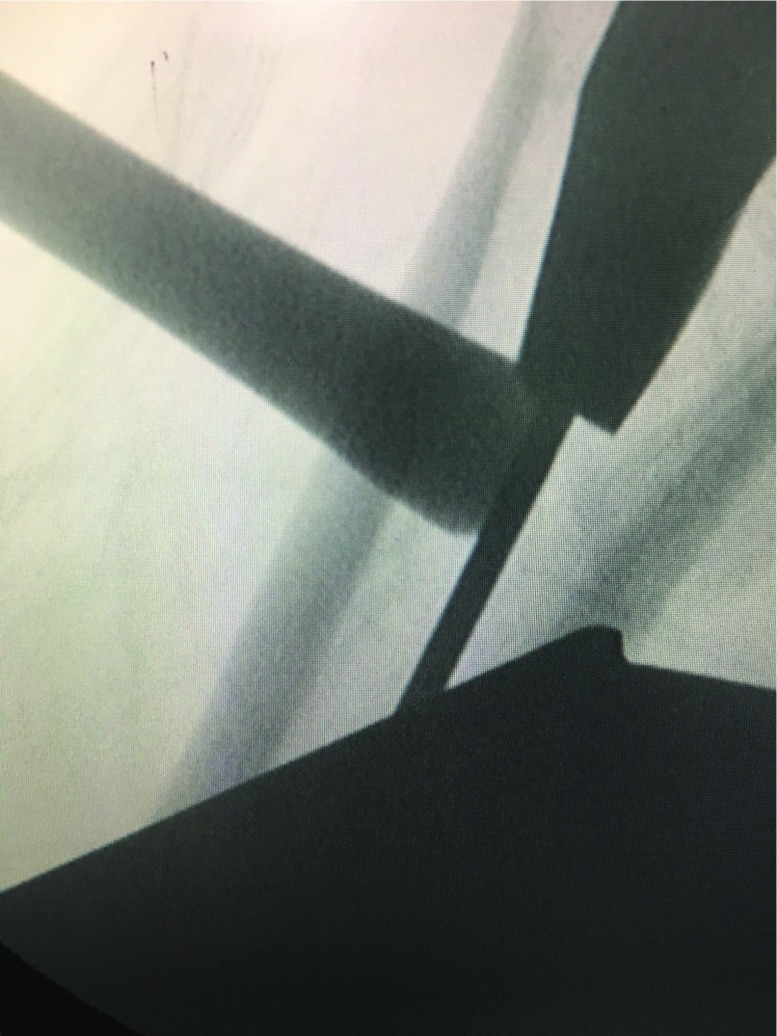
A long strip bony window by using an osteotome, which is just right for inserting a bone detacher.

**Figure 4:**
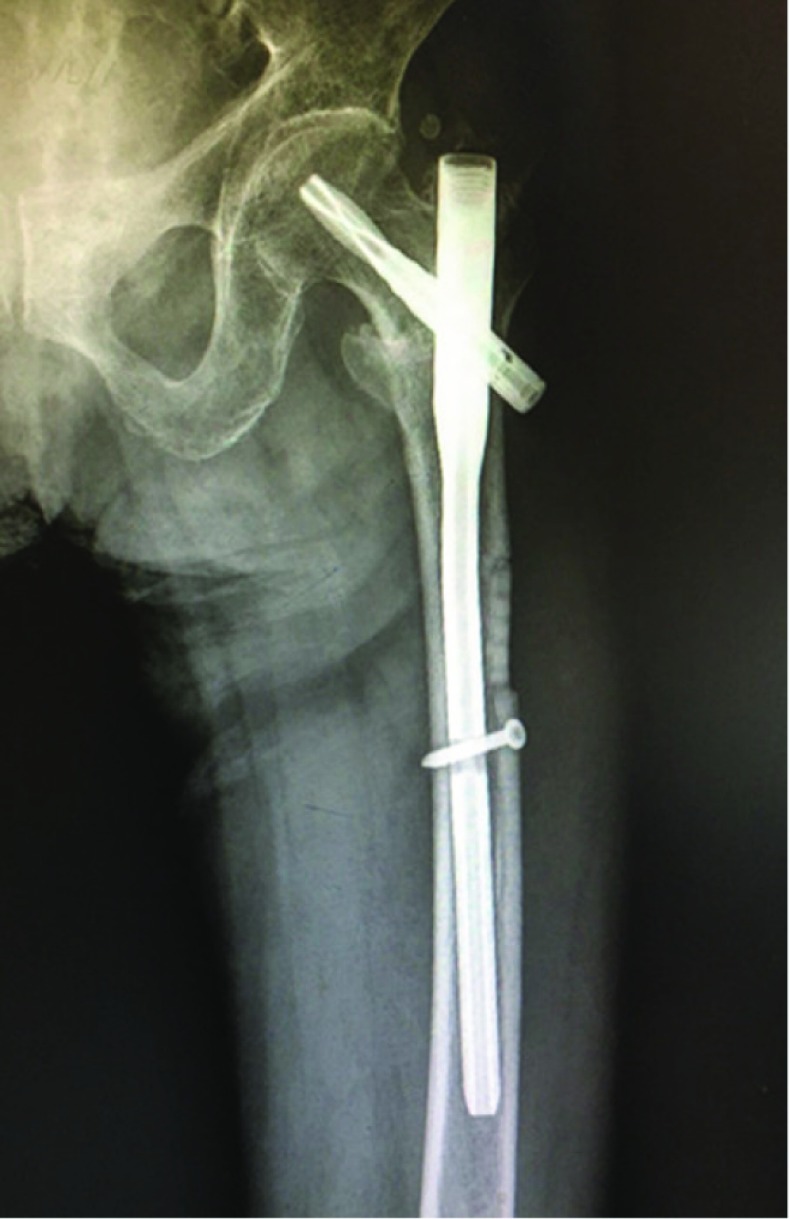
The broken reamer was moved completely, and PFNA was implanted.

The guide needle was removed easily, on account of less experience to refer, and no instrument to use. Therefore we performed an open technique and created a 2.0 cm ×0.4 cm long strip bony window by using an osteotome, which is just right for inserting a bone detacher, then put the detacher head adjoin the reamer, we moved out the broken reamer head by knocking back the inserted detacher and pulling out through the medullary cavity using a Kocher's clamp (Figure 3). A set of PFNA was inserted to fixation and the bony window was full with bony bar taken down before, and the fracture got a good bone union after 2 months (Figure 4).

## Discussion

To the best of our knowledge, no previous cases of incarcerated flexible reamers have been reported in implanting a PFNA. There are several literatures that have been reported for removal of broken femoral nails. Open techniques for nail extraction require opening the fracture or broken site [1], which may lead to bony destruction. Multiple techniques using a fish-hook failed because of the wire had not ample bent to pass through narrow distal tip [2]. And the use of wires as hooks has been reported dangerous as utmost bent as the wire can also break into the nail or medullary cavity [3]. Therefore we could not use a hooked wire to gain purchase into the distal tip [4]. Using double guide wires was difficult because the tapered distal tip of the nail was not adequately wide for a ball tipped wire to pass through [5]. Furthermore, in our case, the reamer was used without a ball-tipped guide wire, and thus, the routine extraction using the guide wire was not possible. De Amorim Cabrita et al. [6] reported an opening technique by using an antegrade reamer to push the distal nail through a drill hole made from a medial parapatellar approach from the knee joint. Other open methods have also been described. Low et al. [7] described a technique for removal of a broken reamer head by drilling with a 4-mm drill at three sites; just distal to the tip of the reamer head, at the middle of the reamer head, and just proximal to the reamer head. The incarcerated flexible reamer was then successfully pulled out [7]. However, as we all know, the reamer for PFNA is much bigger than for common nails, it does not work in this case. Our technique just requires opening a small bony slot. Nevertheless, we acknowledge that creating a cortical window requires a further incision distally which is invasive and does pose the risk of both fracture and non-union at the site of the cortical window. Our cortical window was just 2.0 cm × 0.4 cm and we easily inserted a bone detacher to pull out the broken reamer. We did it with little bone damage, and we replanted the bone strip after pulling out reamer head. In addition, it is important to create the bony window at least 1 cm distal to the tip of the implant to allow space for the detacher insert into the window. Actually, we just need to slack the jammed reamer, when it released from the bone cortical wall, it is very easy to be pulled out. Furthermore, this technique could easily be attempted through a bony window with the handy device, because the detacher was wrapped into every orthopaedic surgical instrument.

This case report reminds us the importance of using a mildly impact force when using a flexible reamer for implanting PFNA. In cases of incarcerated reamers, the long bony slots technique can help to ease the difficult extraction.

## Conclusion

To the best of our knowledge, no previous cases of incarcerated flexible reamers have been reported in implanting a PFNA. Broken reamer jammed into the medullary cavity can provide a difficult challenge for removal. As we experienced, opening a bony slot may settle this trouble easily. Our technique is easy to perform and can potentially be utilised in almost all cases where a broken reamer requires extraction.

## Conflict of interest

The authors declare that they have no conflicts of interest in relation to this article.

## Funding

We have no funding or support to report.
